# Sustainable Carbon Dots from Cellulose Precursors for Environmental Sensing: Recent Trends and Outlook

**DOI:** 10.3390/nano15211649

**Published:** 2025-10-29

**Authors:** Viviana Bressi, Jihene Belhaj, Rayhane Zribi, Ramzi Khiari, Claudia Espro

**Affiliations:** 1 Centro de Estudios para el Desarrollo Sostenible (CEDS), Universidad Tecnológica ECOTEC, Km 13.5 Samborodòn, Guayaquil EC092302, Ecuador; viviana.bressi@unime.it; 2 Dipartimento di Ingegneria, Università di Messina, Contrada di Dio Vill. S. Agata, I-98166 Messina, Italy; jihenebelhadj27@gmail.com (J.B.); razribi@unime.it (R.Z.); 3 Department of Textile, Higher Institute of Technological Studies of Ksar Hellal, Ksar Hellal 5070, Tunisia; khiari_ramzi2000@yahoo.fr; 4 CNRS, Grenoble INP, LGP2, Université Grenoble Alpes, F-38000 Grenoble, France

**Keywords:** carbon dots, cellulose, biomass-derived nanomaterials, green synthesis, environmental sensing, photoluminescence, sustainable materials

## Abstract

Carbon dots (CDs) have emerged as promising nanomaterials for optical sensing due to their outstanding photoluminescence, chemical stability, and biocompatibility. In recent years, the development of sustainable CDs derived from biomass—particularly cellulose—has attracted increasing interest as a green alternative to conventional synthetic routes. This review offers a comprehensive overview of recent advances in synthesis, functionalization, and application of cellulose-based carbon dots for environmental sensing. We examine key synthetic approaches—including hydrothermal, microwave-assisted, and pyrolytic methods—and discuss how the structure and origin of cellulose influence the physicochemical properties of the resulting CDs. The mechanisms underlying their sensing performance are analyzed in detail, with a focus on the detection of heavy metals, organic pollutants, and other environmental contaminants. Challenges related to reproducibility, scalability, and long-term stability are critically addressed. Finally, we outline future directions involving hybrid nanomaterials, real-time sensing platforms, and strategies aligned with circular economy principles. This review aims to serve as a valuable resource for researchers in the fields of sustainable nanomaterials, green chemistry, and environmental sensor development.

## 1. Introduction

In recent decades, growing environmental concerns and the urgency of sustainable development have led both academic and industrial research to increasingly embrace the principles of Green Chemistry. Among its twelve guiding principles, special emphasis is placed on the use of renewable resources, reduction in hazardous substances, and the design of environmentally benign processes. In this framework, the valorization of biomass to produce high-value functional materials has gained strategic importance, offering a viable alternative to conventional fossil-based production models. Among various types of biomass, cellulose—the most abundant, biodegradable, and carbon-rich natural biopolymer—stands out as a particularly attractive precursor for sustainable nanomaterial synthesis [1,2]. At the same time, carbon-based nanomaterials have received increasing attention due to their outstanding structural, optical, and electronic properties, making them suitable for diverse applications in energy, catalysis, biomedicine, and environmental monitoring [3,4]. Carbon dots (CDs), first discovered during electrophoretic separation of single-walled carbon nanotubes in 2004, culminating in the awarding of the 2023 Nobel Prize in Chemistry to this discovery, have emerged as one of the most dynamic classes of carbon-based nanomaterials [5]. These nanoparticles, with a diameter usually less than 10 nm, exhibit remarkable photophysical properties, chemical stability, low cytotoxicity, and versatile surface chemistry, making them highly attractive for applications ranging from environmental monitoring to bioimaging and catalysis [6]. CDs are typically composed of a carbonaceous core, which can be either amorphous or graphitic pattern, surrounded by a shell rich in oxygen-containing functional groups such as hydroxyl (-OH), carboxyl (-COOH), and carbonyl (-C=O). The core–shell structure contributes to their excellent water dispersibility and tunable photoluminescence. Carbon dots (CDs) have gained increasing attention for their strong photoluminescence, low toxicity, and biocompatibility. Their renewable production from biomass precursors also aligns with green chemistry principles, making them attractive for applications in bioimaging, sensing, and photocatalysis. [6,7]. These features, together with their facile surface functionalization, make CDs highly promising candidates for optical sensing, especially in the detection of environmental contaminants such as heavy metals, pesticides, dyes, and other organic pollutants [8,9,10]. However, most conventional CD synthesis methods rely on fossil-based precursors and harsh chemical conditions involving strong acids, oxidants, or toxic solvents—approaches that contradict the principles of sustainability [11]. In response, there has been a rapid expansion of research into green synthesis routes employing biomass-derived carbon sources, particularly cellulose and its derivatives, using environmentally friendly techniques such as hydrothermal treatment, microwave-assisted synthesis, or pyrolysis [12,13,14]. Thanks to its high carbon content and abundance of hydroxyl groups, cellulose facilitates the formation of stable CD structures with favorable photophysical and colloidal properties. The morphology, surface chemistry, and emission properties of the resulting CDs can be finely tuned depending on the type of cellulose (e.g., nanocrystalline, microcrystalline, or native fibers) and the synthetic conditions [3,15]. Recent studies have demonstrated the effectiveness of cellulose-derived CDs in various environmental sensing applications. Zattar et al. synthesized CDs from cellulose via hydrothermal carbonization, functionalized to improve sensitivity to heavy metal ions [16]. Huang et al. reported doped CDs (O, N, S) from cellulose gels capable of detecting Hg^2+^ ions with a detection limit of 0.2 µM [17]. Magagula et al. developed microwave-assisted N-doped CDs from cellulose nanocrystals (CNCs), achieving a detection limit of 75 nM for Fe^3+^ [18]. Sheshmani et al. 2025 produced photocatalytic CDs from cellulose, demonstrating efficient degradation of textile dyes under UV light [7]. Liu et al. 2020 obtained B/N/S-doped CDs from carboxymethylated cellulose, applicable for both Hg^2+^ detection and anti-counterfeiting inks [19]. In this context, the present review provides a comprehensive overview of recent advances in the synthesis, functionalization, and application of cellulose-derived carbon dots for environmental sensing. We highlight how different synthetic methods and cellulose sources influence the structural and optical properties of CDs, and how these properties determine sensing performance. Current challenges—including reproducibility, scalability, and integration into real-time sensing platforms—are critically addressed. Finally, we present future perspectives focused on hybrid nanostructures, low-cost sensor development, and circular economy strategies for biomass valorization.

## 2. Cellulose as a Precursor for Sustainable CDs

Lignocellulosic biomass, including plant stems, grasses, leaves and agricultural residues represents a widely available, low-cost and renewable resource with vast potential for sustainable material development. Among its components, cellulose stands out as the most abundant natural polymer, offering a reliable and eco-friendly feedstock for advanced applications. Its intrinsic properties, such as excellent thermal stability, high tensile strength, and modifiable hydroxyl groups, make cellulose and its derivatives ideal candidates for functionalization and integration into diverse technological domains [20]. Nanocellulose, with its high surface area and outstanding mechanical performance, has catalyzed innovation in the fabrication of biodegradable films, hydrogels, elastomers and microspheres. These materials now play crucial roles in sectors ranging from energy storage to biomedicine and smart manufacturing [21]. Furthermore, cellulose’s bio-based origin, biocompatibility and superior fire behavior compared to synthetic fibers make it an attractive reinforcement in composite materials. This aligns with growing efforts to transition toward a circular bioeconomy. As such, the exploration of cellulose as a precursor for carbon dot (CD) synthesis not only exemplifies the valorization of renewable biomass but also advances the development of high-performance, sustainable nanomaterials [1,22].

### 2.1. Types and Sources of Cellulose

**Figure 1:**
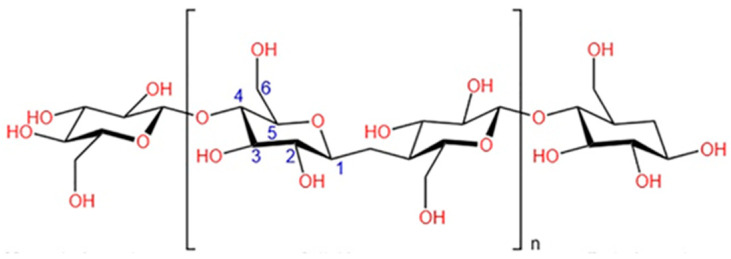
The cellulose macromolecule (adapted from ref. [26] with the permission).

Cellulose was discovered as a chemical substance by Anselme Payen in 1838. He suggested that plant cells are almost all composed of the same fibrous substance that resists treatments with ammonia or acid. Cellulose structure, morphology and chemical composition have remained unknown for a very long time, even though this material has been used for centuries in very diverse applications. The main constituent of photosynthetic organisms is this long polysaccharide, which protects plant organisms [23]. The elemental composition of the cellulose macromolecules is distributed as 49.4% oxygen, 44.4% carbon and 6.2% hydrogen [24]. It is built of linear macromolecules formed by the sequence of cellobiose units. The latter is composed of a sequence of D-glucopyranose units having free OH groups and linked together by β-(1–4) glycosidic bonds (Figure 1), being proposed its general formula as (C_6_H_10_O_5_)n [25,26]. Certain chemical polarity is conferred on native cellulose because of the regularity of the assembly of cellobiose molecules. Once the two ends of cellulose have different chemical functionality, this molecule is provided with a relative polarity with a non-reducing end at the C4 position and a reducing end at the C1 position due to a hemiacetal function of the terminal secondary alcohol.

The principal categories and sources of cellulose employed in sustainable material synthesis encompass microcrystalline cellulose (MCC), nanocellulose and lignocellulosic waste biomass.

#### 2.1.1. Microcrystalline Cellulose (MCC)

Microcrystalline cellulose (MCC) is a highly refined, partially depolymerized form of cellulose obtained through controlled acid hydrolysis of high-grade cellulose sources such as wood pulp or cotton linters [1]. This process selectively removes the amorphous regions of the cellulose polymer, yielding a material composed predominantly of crystalline microstructures. Characterized by its well-defined crystallinity and micrometer-scale particle size, MCC exhibits superior physicochemical properties, including excellent flowability, compressibility, and structural stability [27]. These attributes have established MCC as a critical excipient in pharmaceutical and food industries, where it functions as a binder, filler and disintegrant. Moreover, due to its chemical inertness, high biocompatibility and significant surface area, MCC has gained increasing relevance in advanced material science, particularly in the development of sustainable nanomaterials and drug delivery platforms [28].

#### 2.1.2. Nanocellulose

Nanocellulose refers to cellulose materials engineered at the nanoscale, encompassing cellulose nanocrystals (CNCs), cellulose nanofibers (CNFs) and bacterial nanocellulose (BNC). The two first categories CNC and CNF, are produced by disintegration of cellulose fibers into nanoscale particles below 100 nanometers [29], however, in the case of BNC, the dissolved cellulose is generated by bacteria. The present review is focused on cellulose nanofibers and nanocrystals, specifying each time the extraction processes and the main characteristics. Cellulose Nanocrystals, also known as Cellulose Whiskers (CNCs), are highly crystalline, rod-like nanoparticles produced primarily by acid hydrolysis [30]. The acid hydrolysis process consists of two steps: the alkaline treatment of cellulosic pulp to remove polysaccharide, followed by heat treatment in acid medium for several hours to destroy the amorphous zone more easily and cellulose fiber regions become accessible. At the appropriate point in this step, the acid mixture is spent and residual acids and impurities can be completely removed by repeated centrifugation and dialysis to obtain purified and highly crystalline CNCs [31]. Morphologically, CNCs exist in rod-like or needle-like structures when viewed under a Scanning Electron Microscopy (SEM). The structure, properties, phase separation behavior and dimensions, including length and diameter, of these CNCs vary depending on the type of raw material, acid, hydrolysis temperature, time and the intensity of ultrasonic irradiation [32]. For example, CNCs with a diameter of 200–300 nm and a length of 10–30 nm were obtained when cotton fibers were hydrolyzed by sulfuric acid (H_2_SO_4_) at 65 °C. Cellulose nanofibers (CNFs), in contrast are longer flexible fibrils obtained via three types of processes: (1) mechanical treatments (e.g., homogenization, milling and milling), (2) chemical treatments (e.g., TEMPO oxidation) and (3) a combination of chemical and mechanical treatments [33]. The preparation of CNFs is very simple, involving the physical separation of cellulose fibers by grinding, homogenization, ultrasonication, etc., and requires essentially no modification of the chemical structure of the cellulosic chain [34,35]. Besides the physical process, CNFs can also be produced by chemical methods like oxidation of the raw material by TEMPO under magnetic stirring [36]. Additionally, physical and chemical processes can be used together for the preparation of the individual CNFs, for example, carboxymethylation and high pressure homogenization produce uniformly distributed CNFs [37]. Compared to the formation of hydrogel-based CNCs by self-aggregation, CNF-based hydrogels are formed by the mechanical treatment of their aqueous suspensions with alkalis followed by neutralization [38,39]. Nanocellulose materials exhibit remarkable physicochemical properties, including high tensile strength, biodegradability, tunable surface chemistry via abundant hydroxyl groups, and excellent biocompatibility, making them attractive for diverse applications such as biomedical devices, composites, electronics, and sustainable precursors for carbon nanomaterials like carbon dots.

#### 2.1.3. Waste Biomass

**Figure 2:**
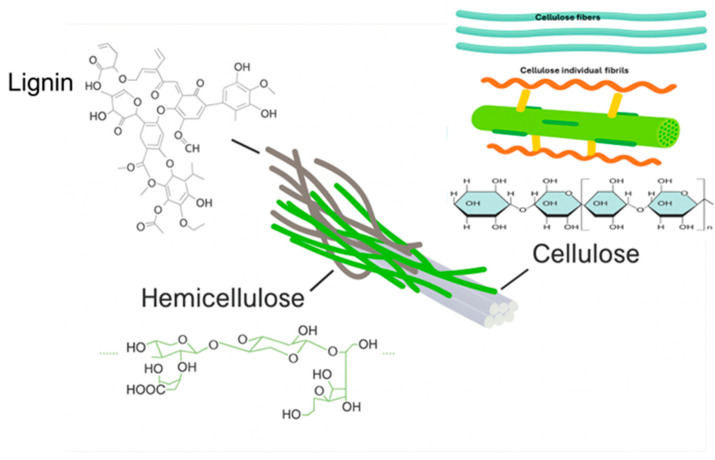
Schematic Representation of Cellulose, Hemicellulose and Lignin Components. (Adapted from ref. [46] with the permission).

Lignocellulose constitutes the fundamental structural component of plant cell walls. Plant biomass predominantly comprises cellulose, hemicellulose and lignin, accompanied by smaller proportions of pectin, proteins and extractives such as nonstructural sugars, chlorophyll and waxes as well as ash [40]. The relative composition of these constituents varies significantly among different plant species for instance, hardwood typically contains a higher cellulose content, whereas wheat straw and foliage are richer in hemicellulose [41]. Furthermore, the proportions of these components within a single plant species depending on factors such as age, developmental stage and environmental conditions [42]. The Cellulose is a linear homopolysaccharide, forming highly ordered crystalline microfibrils that provide structural strength and rigidity to plant cell walls [43]. Hemicelluloses are heterogeneous branched polysaccharides such as xylans, mannans, and glucans that surround cellulose microfibrils and contribute to flexibility and moisture [44]. Lignin is a complex aromatic polymer derived from phenylpropanoid monomers like coniferyl and sinapyl as shown in Figure 2, imparts hydrophobicity, rigidity and resistance to enzymatic degradation [45].

**Figure 3:**
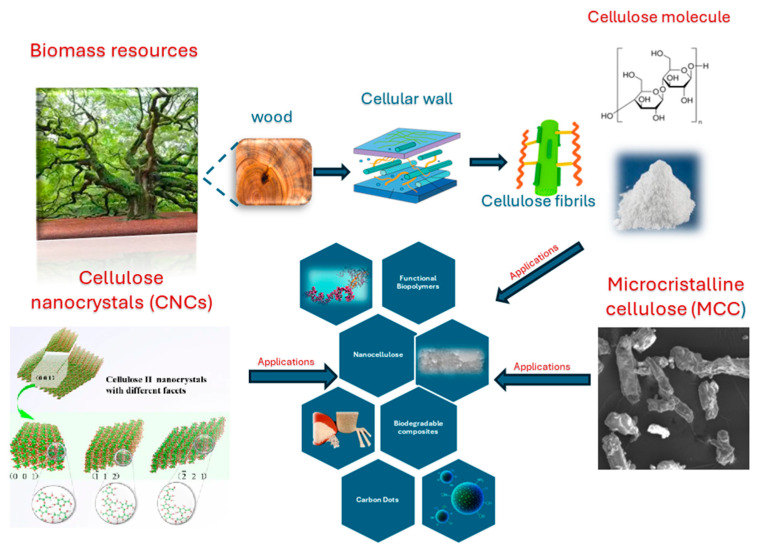
Sustainable applications from different cellulose sources (CNCs, MCC, and lignocellulosic biomass). (Adapted from refs. [28,30,42] with the permission).

Advances in eco friendly pretreatment and fractionation technologies such as physical milling, steam explosion and mild chemical treatments have enabled more efficient separation of these components, particularly facilitating the recovery of high-purity cellulose from complex biomass matrices [46]. The extracted cellulose is increasingly viewed as a versatile precursor for sustainable applications, including nanocellulose fabrication, biodegradable composites, biofuels and functional biopolymers. Thus, as schematically represented in Figure 3, valorizing lignocellulosic residues aligns strongly with circular bioeconomy strategies by transforming low-value waste into functional, high-performance materials with broad industrial utility.

### 2.2. Structural Features Influencing the Carbonization of Cellulose

Cellulose is structural characteristics critically influence its carbonization behavior, directly impacting the physicochemical properties of the resulting carbonaceous materials. Among the most influential features are crystallinity, functional group composition and thermal decomposition dynamics. Cellulose is composed of linear chains as we mentioned before, which form highly ordered crystalline and amorphous regions stabilized by extensive intra- and intermolecular hydrogen bonding. These crystalline domains enhance thermal resistance and dictate the decomposition pathway under pyrolytic conditions [47]. Nanostructured forms as microcrystalline cellulose (MCC) exhibit elevated crystallinity and thermal stability, resulting in distinct carbonization profiles compared to amorphous cellulose types [48]. During heating, cellulose undergoes a series of thermally induced transitions. At temperatures between 250–400 °C, initial depolymerization and dehydration occur, driven largely by the breakdown of hydroxyl-rich glycosidic bonds, which release volatile compounds and form reactive intermediates [49]. This is followed by the formation of conjugated sp^2^ carbon clusters in the 450–600 °C range and further structural reorganization into curved lamellae and partially graphitized domains between 650–1000 °C [50]. At temperatures exceeding 1000 °C, these aggregates further evolve into stable, highly conductive graphitic structures characterized by sp^2^ hybridization and low electrical resistivity. The supramolecular hydrogen bonding network not only influences thermal degradation but also governs how cellulose chains rearrange and crosslink during carbonization, thereby affecting carbon yield and nano structural morphology [51]. Notably, nanocellulose, due to its high specific surface area and enhanced reactivity, enables precise control over the shape, size and conductivity of carbon nanomaterials such as carbon dots [51]. Collectively, these structural features including crystallinity, functional group reactivity, hydrogen bonding architecture and nanostructure determine the tunability of carbon materials derived from cellulose. In summary, the structural and compositional characteristics of cellulose, such as its crystallinity, hydrogen-bonding network, and functional group distribution, play a decisive role in governing its carbonization dynamics and the structure of the resulting carbon materials. These parameters not only determine the yield and morphology of the produced carbonaceous structures but also influence their optical and surface properties. Understanding these correlations is fundamental for evaluating the suitability of cellulose as a precursor for carbon dots and for identifying the key advantages and limitations associated with its use, as discussed in the following section.

### 2.3. Advantages and Limitations of Cellulose as a Precursor for Carbon Dots

Cellulose, the most abundant biopolymer on Earth, presents a promising and sustainable precursor for the synthesis of carbon-based nanomaterials, particularly carbon dots (CDs). Readily available from plant matter and diverse biomass waste, cellulose is aligned with circular bioeconomy principles due to its renewability and production by rapidly growing, carbon-sequestering organisms. Its molecular structure rich in hydroxyl, carboxyl and aldehyde groups confers high chemical reactivity, facilitating heteroatom doping and surface functionalization. These features enable precise modulation of physicochemical properties such as fluorescence, hydrophilicity and biocompatibility, thereby expanding the utility of cellulose-derived CDs in biomedical diagnostics, catalysis and environmental applications [26,42]. However, practical challenges remain, including cellulose’s relatively low carbon content (~44%), variability in composition due to the presence of lignin and hemicellulose and the complexity of extraction and purification processes. Addressing these limitations through optimized pretreatment and controlled carbonization is crucial to enhancing process efficiency and reproducibility in the sustainable fabrication of high-performance nanocarbon materials [52]. A summary of these characteristics and challenges is presented in Table 1.

Building on the understanding of cellulose’s potential as a renewable precursor, the next section examines how various synthesis routes translate these structural and chemical features into carbon dots with tailored morphology, photoluminescence, and functionality.

## 3. Synthesis Strategies

**Figure 4:**
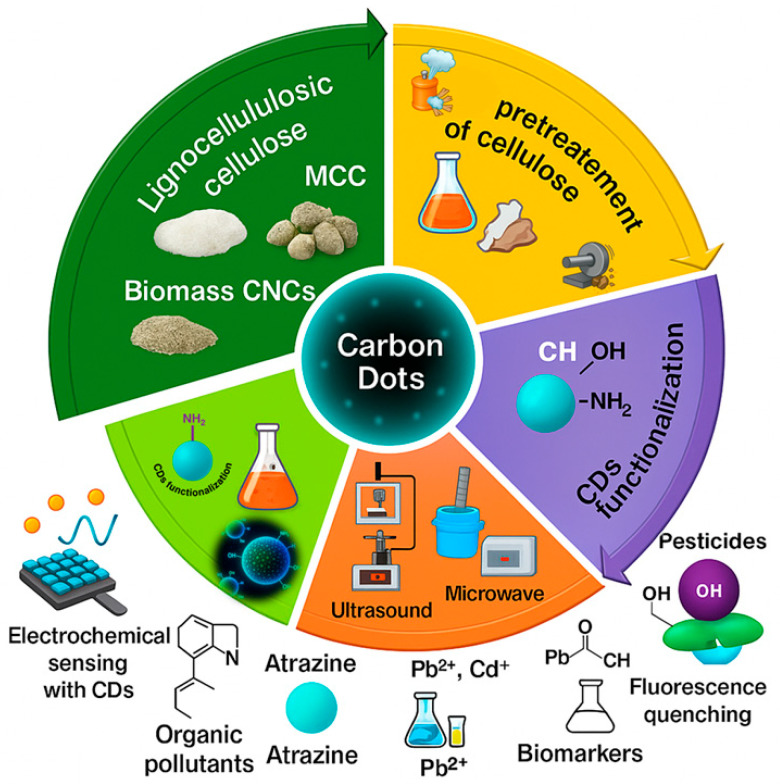
Schematic overview of cellulose-derived carbon dots (CDs) synthesis and sensing applications. From left: lignocellulosic raw materials (e.g., wood pulp, cotton, agricultural residues) are converted into MCC (microcrystalline cellulose) and CNCs (cellulose nanocrystals). On the right, the employed synthesis methods include hydrothermal (180–250 °C, 4–12 h), pyrolysis (300–500 °C), solvothermal (160–200 °C, 6–10 h), microwave (120–200 °C, 10–30 min), and ultrasound (RT, 30–60 min). In the center, the resulting carbon dots (small fluorescent spheres) are linked to their sensing applications: heavy metals (Pb^2+^, Cd^2+^), pesticides (atrazine, carbendazim), organic pollutants (phenol), and biomarkers (dopamine, glucose).

The synthesis of carbon dots from cellulose precursors leverages Green Chemistry principles by utilizing abundant, renewable biomass sources. Several synthetic routes have been developed to convert cellulose, ranging from microcrystalline cellulose to nanocellulose and agro-industrial waste, into CDs. As schematically shown in Figure 4, the main approaches include hydrothermal, microwave-assisted, pyrolysis, ultrasound-assisted, and other emerging green techniques.

### 3.1. Hydrothermal and Solvothermal Methods

Hydrothermal synthesis remains one of the most employed techniques for preparing cellulose-derived CDs. This process involves heating an aqueous suspension of cellulose in a range of temperatures typically from 150 up to 300 °C, in a sealed autoclave, leading to hydrolysis, carbonization, and passivation in a single step [59]. Parameters such as reaction time, temperature, and precursor concentration significantly influence the size, surface chemistry, and quantum yield of the resulting CDs [60,61]. In a very recent study, pure cellulose was autoclaved at high temperature and pressure to form CQDs, which were subsequently anchored to form heterojunctions with g-C_3_N_4_, enhancing the photocatalytic degradation of lignin [62]. The hydrothermal process enables the direct synthesis of pre-doped carbon nanomaterials, either through the addition of suitable dopant precursors during the reaction or by exploiting the intrinsic heteroatom content of the starting biomass. For instance, Bavya et al. 2025 reported the one-pot synthesis of S-doped carbon dots from cellulose, where sulfur doping enhanced the sensing performance of CDs [63]. Rani et al. employed a solvothermal approach in a 25 vol% ethanol–water mixture, using curcumin as a non-aqueous carbon precursor, yielding highly hydrophilic CDs suitable for bioimaging applications [64].

### 3.2. Microwave-Assisted Synthesis

Microwave irradiation offers rapid, energy-efficient CD synthesis by inducing uniform heating and accelerating carbonization. Cellulose precursors, often pre-treated with mild acids or alkaline agents, are exposed to microwave radiation (typically 300–900 W) for short durations (5–20 min), resulting in CDs with high quantum yield and narrow size distribution [65,66]. Recent studies have further demonstrated that cellulose-based feedstocks are highly suitable for microwave-assisted approaches. Hasan et al. reported the microwave-assisted synthesis of N-doped CDs from carboxymethylcellulose and glycine, where modulation of microwave power directly influenced particle size uniformity and surface functionalization, leading to a quantum yield of ~31% and enhanced ion-sensing performance [67]. Similarly, Singh et al. demonstrated that biomass-derived S- and N-doped CDs incorporated into cellulose nanocrystal films exhibited tunable photoluminescence properties, strongly dependent on irradiation parameters such as power intensity and exposure time [68]. Moreover, Lee et al. highlighted that operational parameters including temperature ramp rate and irradiation cycles significantly impacted the antioxidant and antimicrobial activity of lignocellulose-derived CDs [69].

### 3.3. Pyrolysis and Combustion Techniques

Dry pyrolysis involves the thermal decomposition of cellulose in inert atmospheres (e.g., N_2_ or Ar) at temperatures between 300–600 °C. This technique yields CDs with high carbonization degrees but often requires subsequent purification steps to remove graphitic debris. The combustion method, by contrast, employs open-air burning of cellulose, generating CDs with oxygen-rich surfaces, albeit with lower quantum yield and reproducibility. Pyrolysis of nanocrystalline cellulose at 300 °C resulted in nitrogen-doped CDs with a quantum yield of 29%, suitable for Hg^2+^ detection. Similarly, a solvent-free pyrolysis strategy using biomass components like cellulose and lignin yielded CDs with quantum yields of 11.7% and 23.4%, respectively [70,71]. Compared to hydrothermal and microwave-assisted methods, pyrolysis offers advantages such as scalability, solvent-free processing, and high carbonization efficiency. However, it also presents challenges, including higher operational temperatures, lower quantum yields, potential for graphitic impurities, and the need for post-synthesis purification.

### 3.4. Ultrasound-Assisted and Other Green Methods

Ultrasound irradiation generates cavitation bubbles that induce localized high temperatures and pressures, promoting cellulose degradation and CD formation. This approach often involves co-solvents or mild oxidants, yielding CDs with abundant surface functionalities, high QY, and good photostability [72]. Recent advances have introduced novel green approaches such as deep eutectic solvent (DES)-assisted and microfluidic syntheses of carbon dots (CDs). DESs have emerged as sustainable reaction media due to their low volatility, biodegradability, and tunable hydrogen-bonding networks that can facilitate carbonization at lower temperatures. The recent study by Tang et al. introduced a novel approach for synthesizing biomass-derived CDs using DES under mild conditions [73]. This method processed lignocellulose at 110 °C, yielding CDs with strong fluorescence (360–550 nm emission) and enabling DES recycling. The CDs demonstrated excellent potential for Fe^3+^ detection (LOD = 191 ppb), stable optical properties, and versatility in inks and flexible composite films. Compared to other methods such as pyrolysis or hydrothermal treatment, ultrasound offers advantages including shorter reaction times, lower energy consumption, and operation at near-ambient temperatures. However, the optical quality of CDs produced by ultrasound can be lower than those obtained via pyrolysis, which typically achieve higher quantum yields but require harsher conditions and risk generating impurities [74]. In parallel, microfluidic synthesis has attracted increasing attention for its precise control over reaction parameters, efficient heat and mass transfer, and potential scalability compared with batch methods. Although microfluidic platforms are still rarely applied to cellulose-derived CDs, they offer continuous and reproducible processing, which could substantially improve particle size homogeneity and optical tunability. Comparing these emerging routes to conventional hydrothermal, microwave, or ultrasound-assisted methods, DES-based and microfluidic syntheses exhibit clear environmental and scalability advantages. DES systems minimize solvent waste and allow partial recyclability, though recent reports caution about possible degradation of certain DESs such as ethaline under ambient conditions. Microfluidic reactors, on the other hand, significantly reduce energy consumption and reaction time, aligning well with green chemistry principles. Overall, the integration of DES and microfluidic strategies represents a promising step toward next-generation, sustainable, and industrially scalable CD production [73,74]. A comparative overview of these methods is provided in Table 2.

As shown, hydrothermal and microwave-assisted methods offer an attractive balance between quantum yield and environmental sustainability. Furthermore, the type of cellulose precursor critically influences product morphology and optical features. For instance, nanocellulose tends to yield CDs with narrower size distributions and enhanced fluorescence due to its high surface area and reactivity. The transformation of cellulose into carbon dots involves complex dissolution, depolymerization, and carbonization mechanisms, which are strongly influenced by the solvent system and reaction conditions. According to a recent study [22] partial dissolution of cellulose in deep eutectic or ionic liquid systems enhances the accessibility of β(1–4) glycosidic linkages, promoting their cleavage during the early stages of carbonization. This controlled depolymerization facilitates the formation of short-chain oligosaccharides that subsequently undergo dehydration and aromatization to yield furanoid intermediates such as 5-hydroxymethylfurfural (HMF) and related polyfurans. These intermediates undergo further condensation, cross-linking, and aromatization reactions, gradually evolving into disordered graphitic nuclei that constitute the carbon core of CDs. The residual oxygenated surface groups (–OH, –COOH, –C=O) originate from incomplete dehydration or oxidation processes, accounting for the strong photoluminescence and hydrophilicity of the resulting CDs. The solvent environment plays a critical role in directing these reactions: for instance, DES or other hydrogen-bond-rich systems stabilize reactive intermediates and control the rate of dehydration and polymerization, thereby modulating the size and emission properties of CDs. These findings highlight how tailoring cellulose dissolution and early-stage carbonization can provide a route toward better-controlled and more environmentally friendly CD synthesis from renewable biomass. In summary, each synthesis strategy offers a distinct balance between sustainability, efficiency, and control over the structural and optical features of cellulose-derived carbon dots. Hydrothermal and microwave-assisted methods generally provide uniform particle sizes and high quantum yields but require elevated temperatures or pressures and may face scalability challenges. Pyrolysis and combustion routes are cost-effective and easily scalable but often result in heterogeneous products with lower photoluminescence efficiency. Ultrasound-assisted and other emerging green methods, such as deep eutectic solvent or enzymatic approaches, stand out for their mild operating conditions and environmental compatibility, although they still need optimization and standardization. These advantages and limitations directly influence the physicochemical properties, fluorescence performance, and sensing behaviour of the resulting CDs. Understanding how synthesis conditions affect carbon core structure, surface functionality, and defect density is thus crucial for tailoring CDs toward specific applications, ranging from environmental monitoring and pollutant detection to photocatalysis and bioimaging.

## 4. Carbon Dots Properties Relevant to Sensing and Sensing Mechanisms

Based on synthesis route and structure, CDs are generally categorized into three main groups: carbon nanodots (CNDs), carbon quantum dots (CQDs), and graphene quantum dots (GQDs). CNDs are amorphous and lack a defined lattice structure, CQDs exhibit quantum confinement and edge effects due to their nanocrystalline sp2 carbon core, and GQDs possess layered graphitic domains with clear graphene lattice fringes [75,77,78,79,80]. One of the most remarkable features of CDs is their excitation-dependent photoluminescence (PL). This phenomenon, wherein the emission wavelength changes with excitation wavelength, is attributed to several factors: (i) quantum size effect, where smaller dots exhibit blue-shifted emission; (ii) surface state emissions due to heteroatoms or surface defects; and (iii) molecular state emissions from fluorophores on the surface. In many cellulose-derived CDs, the photoluminescence arises primarily from surface states and carbon core transitions [81]. The quantum yield (QY) of CDs, which is an indicator of optical efficiency, varies significantly with synthesis method, precursor material, and surface treatment. In green synthesis routes, such as from cellulose raw materials, QY values may range from 5% to 35%, and can be improved through doping with heteroatoms like nitrogen or sulfur, or surface passivation with agents like polyethylene glycol (PEG) or citric acid. Apart from their optical behavior, CDs demonstrate favorable electron transfer properties, electrochemical activity, and high surface-to-volume ratios, which are particularly relevant for sensing applications. Their nanoscale dimensions enable facile diffusion and interaction with target analytes, while their rich surface chemistry allows for functionalization with selective ligands or receptor molecules. Biocompatibility and low toxicity are also key attributes, particularly when CDs are synthesized from biomass such as cellulose, lignin, or starch. This makes them ideal candidates for environmental sensing, where they can detect contaminants in water or soil with minimal ecological impact.

**Figure 5:**
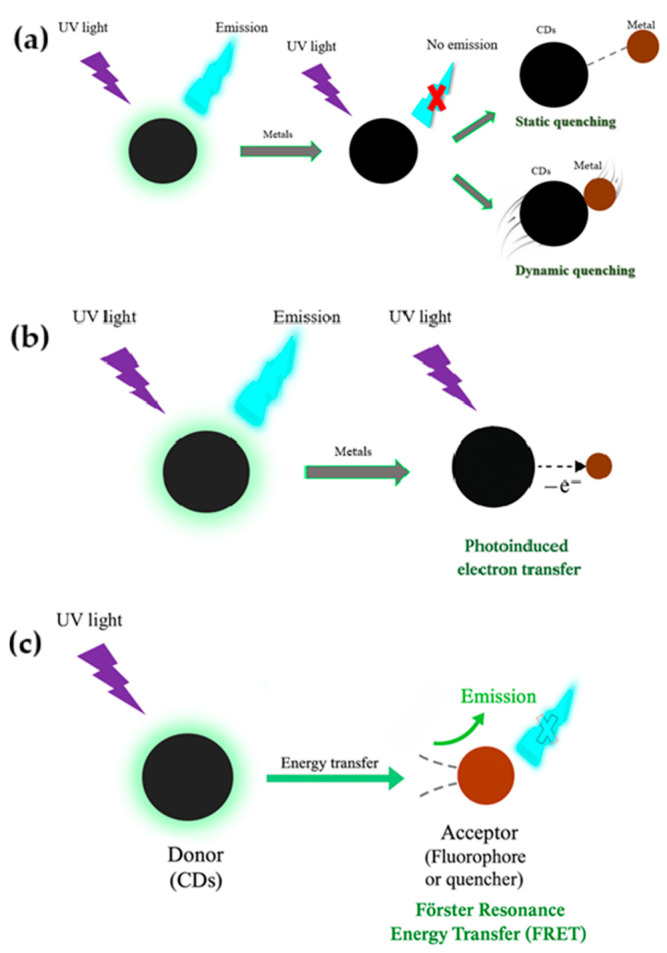
Schematic representation of quenching mechanisms and energy exchange between CDs and various analytes. (**a**) fluorescence quenching; (**b**) photo-induced electron transfer; (**c**) Forster Resonance energy transfer.

As reported in Figure 5, in the context of sensing, several mechanisms are employed by CDs to convert the interaction with analytes into a detectable signal:

- Fluorescence Quenching: This occurs when the emission of CDs is reduced due to energy or electron transfer between the CDs and the analyte. Common quenchers include heavy metal ions (e.g., Hg^2+^, Fe^3+^), nitroaromatics, or peroxides [82,83].
- Förster Resonance Energy Transfer (FRET): A distance-dependent energy transfer process between a donor (CDs) and an acceptor fluorophore or quencher. Effective FRET requires spectral overlap and physical proximity (<10 nm) [84].
- Inner Filter Effect (IFE): Analytes may absorb the excitation or emission light of CDs, leading to apparent quenching without direct interaction [85].
- Static and Dynamic Quenching: Static quenching involves the formation of a non-fluorescent complex between the CD and analyte, whereas dynamic quenching results from collisional encounters [74].
- Photoinduced Electron Transfer: Occurs when excited-state CDs transfer electrons to an electron-deficient analyte, modulating the emission signal [76].
- Dexter energy transfer (DET): happens when the donor and acceptor molecules are so close that their electron orbitals overlap, allowing an electron exchange. Because this overlap is essential, DET only works at sub-nanometer distances and is typical in tightly packed molecular systems [76].
- Surface energy transfer (SET): occurs when an excited fluorophore interacts with the conduction electrons of a nearby metal surface or nanoparticle. In-stead of orbital overlap, it relies on electromagnetic coupling, and its efficiency decreases with the fourth power of distance, making it important in plasmonic and nanoscale sensors [76].

Static and dynamic quenching, photoinduced electron transfer (PET), and the inner filter effect (IFE) are some of the quenching mechanisms that apply to CDs while Forster resonance energy transfer (FRET), Dexter energy transfer (DET), and Surface energy transfer (SET) are the three types of energy exchange. CDs also exhibit electrochemical responsiveness, which can be exploited in sensor electrodes for current or potential-based detection. Their redox-active surfaces, particularly in doped forms, can catalyze reactions with environmental toxins or facilitate charge transfer processes in electrochemical sensors. The high stability of CDs under UV irradiation, extreme pH, or varying temperatures further supports their practical use. Additionally, CDs can be engineered to emit across the visible to near-infrared spectrum, enabling multiplexed or deep-tissue sensing applications. From a structural viewpoint, CDs synthesized from cellulose or their derivatives often retain some of the native functional groups of the precursor, contributing to hydrophilicity and reactivity. This intrinsic functionality eliminates the need for harsh chemical modifications post-synthesis, aligning with green chemistry principles. For example, CDs derived from nanocellulose show abundant surface -OH groups, which promote hydrogen bonding with analytes and high solubility in water. Recent innovations also include the development of dual-mode sensors combining photoluminescence and electrochemistry, or ratiometric probes where emission intensity at two wavelengths is modulated by the analyte. These advances rely on precise control over the electronic structure of CDs, achievable via surface engineering and dopant incorporation [71,86]. In conclusion, carbon dots present a multifaceted platform for sensing applications due to their tunable optical and electrochemical properties, small size, and compatibility with aqueous systems. When synthesized from sustainable precursors such as cellulose, they offer a low-cost, environmentally friendly, and effective tool for real-time environmental monitoring, with ongoing research pushing the boundaries of sensitivity, selectivity, and multifunctionality. While the intrinsic optical and electronic characteristics of cellulose-derived CDs define their sensing potential, further modulation through chemical or elemental modification can significantly expand their performance range. The following section discusses functionalization strategies aimed at tuning these properties for enhanced selectivity and stability.

## 5. Functionalization and Property Tuning

The surface functionalization of cellulose-derived CDs plays a pivotal role in dictating their photophysical properties and sensing performance. Post-synthetic modifications or in situ doping strategies are often applied to introduce new functionalities, passivate surface defects, and enhance target selectivity. These modifications not only improve optical properties but also tailor CDs for specific applications such as bioimaging, sensing, and environmental monitoring.

### 5.1. Surface Passivation

Surface passivation involves the modification of the CDs’ surface to reduce surface defects and trap states, leading to improved fluorescence and stability. Common passivating agents include polyethylene glycol (PEG), ethylenediamine (EDA), and citric acid. PEG passivation enhances the water solubility and biocompatibility of CDs, making them suitable for biological applications. PEGylated CDs exhibit reduced cytotoxicity and improved stability in physiological conditions. For instance, PEGylated CDs synthesized from citric acid and EDA demonstrated ultra-bright blue-violet luminescence with a quantum yield up to ∼100% [87]. EDA serves as both a nitrogen source and a passivating agent. EDA-modified CDs show significantly stronger blue fluorescence compared to unmodified counterparts due to better passivation of surface trap states [88]. Instead, citric acid can act as a carbon source and, when combined with other agents like EDA, facilitates the formation of CDs with improved optical properties. The presence of carboxyl groups from citric acid contributes to the surface passivation, enhancing fluorescence [89,90].

### 5.2. Elemental Doping

Elemental doping introduces heteroatoms into the carbon core of CDs, modifying their electronic structure and enhancing specific properties. Nitrogen-doped CDs (N-CDs) exhibit improved quantum yields and enhanced electron-donating capabilities, beneficial for sensing electron-deficient analytes. For example, N-CDs synthesized from glucose and ammonia demonstrated a quantum yield of up to 40% [91]. Phosphorus-doped CDs (P-CDs) exhibit increased chemical affinity toward specific metal ions, making them suitable for selective sensing applications. P-CDs prepared via solvothermal synthesis have shown bright green emission and high photostability [92]. Sulfur doping introduces mid-gap states and red-shifts emission, broadening the absorption spectrum and enhancing the CDs’ sensitivity to various analytes. Co-doping with nitrogen and sulfur has been shown to create redox-active sites, improving antioxidant efficacy and sensing performance [93].

### 5.3. Metal Doping and Composite Formation

Metal-doped carbon dots (CDs), incorporating elements such as iron (Fe), copper (Cu), and manganese (Mn), exhibit enhanced catalytic properties and improved signal responses in electrochemical detection systems. The introduction of metal ions into the carbon core modifies the electronic structure, creating additional active sites that facilitate specific interactions with target analytes. Fe-CDs synthesized via a one-step hydrothermal method using o-phenylenediamine and FeCl_3_·6H_2_O demonstrate enhanced orange fluorescence and a red-shifted emission spectrum. These CDs exhibit selective fluorescence quenching in the presence of Cu^2+^ ions, enabling sensitive detection with a limit of detection (LOD) as low as 0.23 µM [94]. Cu-doped CDs have been utilized in dual-mode sensing platforms, combining fluorescence and colorimetric responses for the detection of Cu^2+^ ions. The fluorescence quenching mechanism is attributed to the inner filter effect (IFE) between the chromogenic product and the CDs. This dual-response system offers enhanced sensitivity and selectivity for Cu^2+^ detection in complex matrices [95].

### 5.4. Tailoring for Target-Specific Sensing

**Figure 6:**
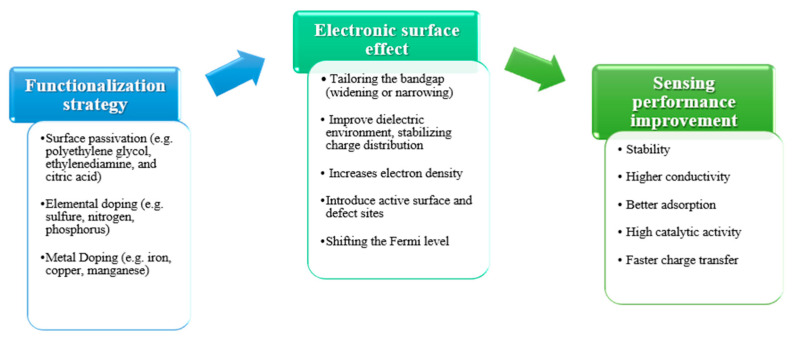
Effect of functionalization on the electronic structure and sensing performance of CDs.

Functionalizing the surface of CDs with specific chemical groups enhances their affinity and selectivity toward target analytes. Figure 6 below illustrates a schematic diagram that depicts the impact of the functionalization strategy on the enhancement of sensing performance through the modelling of the electronic structure of CDs.

Surface modifications such as amine (-NH_2_), thiol (-SH), and carboxyl (-COOH) groups enable CDs to interact with multiple analytes through mechanisms like chelation, hydrogen bonding, and electrostatic interactions. Alkyl amine-functionalized CDs demonstrate selective detection of nitroaromatic compounds, such as dinitrophenol and dinitroaniline, through fluorescence quenching and colorimetric changes [96]. CDs functionalized with thiol groups exhibit high selectivity for mercury ions (Hg^2+^) due to the strong chelation between the thiol groups and Hg^2+^. This interaction leads to significant fluorescence quenching, allowing for ultrasensitive detection of Hg^2+^ with a detection limit of 6.8 nM [97]. Carboxylated CDs interact with organic amines and nitro compounds through hydrogen bonding and electrostatic interactions, enabling selective detection of these analytes. The presence of carboxyl groups on the surface of CDs enhances their water solubility and biocompatibility, making them suitable for environmental and biological sensing applications [98,99]. Recent research also explores dual or multi-element doping to synergistically enhance properties. For instance, N, S co-doped CDs exhibit both improved fluorescence and higher binding affinity for metal ions, facilitating ratiometric or multiplexed sensing strategies [93].

These results illustrated in Table 3, suggest that strategic functionalization of cellulose-derived CDs is crucial for fine-tuning their electrochemical responses and expanding their sensing repertoire. Overall, functionalization strategies play a decisive role in bridging the intrinsic properties of cellulose-derived CDs with their practical applications. By tailoring surface chemistry, doping, and composite formation, it becomes possible to fine-tune fluorescence behaviour, selectivity, and stability. These improvements are directly reflected in the sensing performance of CDs, as discussed in the following section focused on their environmental applications.

## 6. Environmental Sensing Applications

Recently, chemical sensors were the type of sensors that are widely used. Their working principle consists of taking the chemical property change caused by interaction with an analyte and converting it into an electrical or optical signal that can be quantitatively measured, enabling detection and quantification of chemical substances. This approach offers several major advantages, including high sensitivity, adjustable selectivity, a short response time, and portability, which facilitates their use in the field. They are currently the subject of many research and development in the field of environmental and agricultural analyses. Such devices occupy a leading position among the currently available sensors which are already at the commercial stage. In the context of environmental detection, chemical sensors based on cellulose derived carbon dots are particularly suited to the identification of trace contaminants in water, air, or soil. They enable real-time monitoring of environmental quality, provide early warning of pollution, and contribute to the protection of public health. Their versatility is demonstrated by the diversity of analytes detected, ranging from toxic heavy metals to organic pollutants, including anions and small molecules of environmental interest. In this section, the significant recent advances in the design of CDs from ecofriendly synthetic routes, for the development of electrochemical sensors for different analytes, are summarized with a comparative and balanced discussion (Table 4).

### 6.1. Detection of Heavy Metals

**Figure 7:**
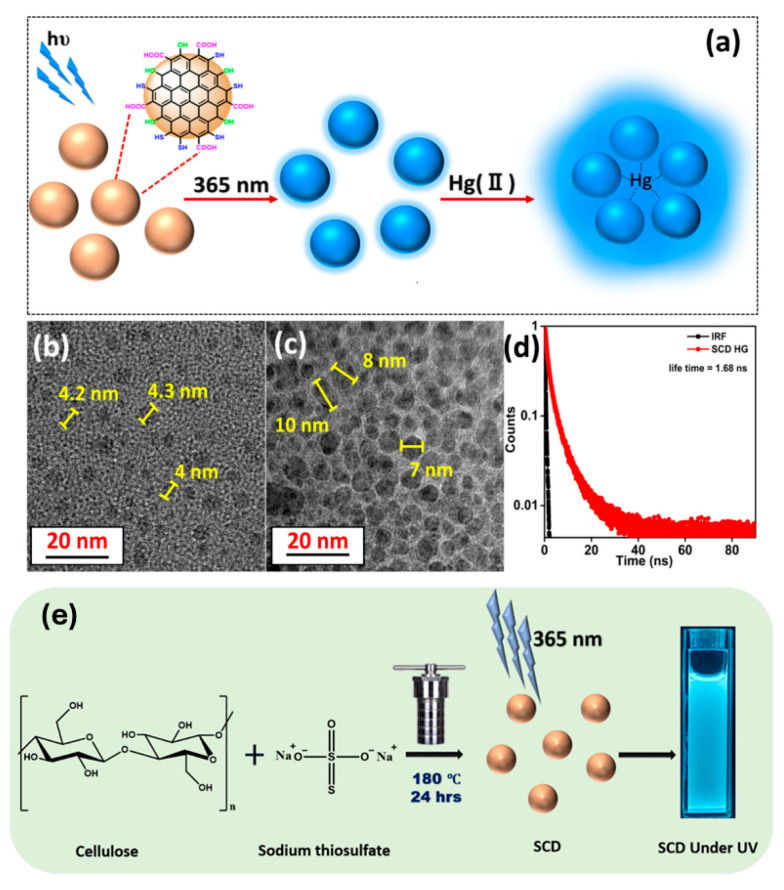
Synthesis of SCD from Bamboo Cellulose via hydrothermal synthesis employing cellulose as the carbon source and sodium thiosulfate as the passivating agent (**a**). Morphological analysis of SCD: (**b**) TEM images of SCD at 20 nm scale, (**c**) SCD after the addition of Hg (II), (**d**) fluorescence lifetime of the SCD-Hg (II) solution measured using the TCSPC technique, and (**e**) schematic representation of SCDs hydrothermal synthesis (180 °C, 24 h) and the subsequent luminescent behaviour under 365 nm UV irradiation. Adapted with permission from the ref. [63].

Huang et al. presented green, cost-effective route to produce biocompatible fluorescent carbon dots from cellulose hydrogel for the specific detection of mercury (Hg^2+^). Starting from the cellulose hydrogel, which is a natural polymer, CDs were prepared via hydrothermal process using different pretreatments method to obtain particles with different size ranging from 2.11 nm to 8.72 nm followed by a doping step with oxygen, nitrogen, and sulfur to enhance their optical and sensing properties. The smallest CDs doped with nitrogen presented a strong fluorescence quenching in presence of Hg2 due to the surface complexation between Hg^2+^ and functional groups on the CDs, and was extremely selective even in presence of other interferent analytes such as Fe^3+^, Zn^2+^, Mg^2+^, Ni^2+^, etc. The proposed sensor exhibit two linear ranges from 0.2–10 μM and 10–100 μM with a limit of detection around 0.2 µM [17]. In addition, the group of Huang, Xiaoning et al. presented multifunctional carbon dots derived from carboxymethyl nanocellulose prepared via hydrothermal method and doped with different elements for the detection of mercury. All the prepared CDs showed blue emission under UV excitation, but different fluorescence behavior was detected according to the doping element. Notably, the amine-modified CDs exhibited the highest fluorescence quenching upon exposure to Hg^2+^ with a low limit of detection of 8.29 × 10^−6^ mol/L compared with other functional group modified CDs and showed excellent selectivity against competing ions [100]. In contrast to the previously mentioned studies, Bavya et al. 2025 presented a turn-on fluorescence mode differing from the more common quenching approach upon exposure to Hg^2+^ via bamboo cellulose-derived carbon dots [63]. Main results obtained by Bavya et al. are reported in Figure 7.

**Figure 8:**
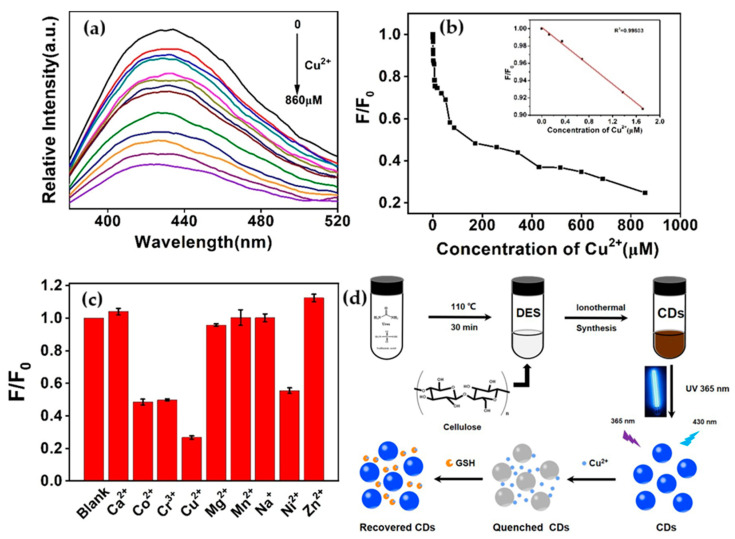
Sensitivity of Cu^2+^ detection. (**a**) Fluorescence intensity of CDs with different Cu^2+^ concentrations from 0 to 860 μM; (**b**) The linear relationship between F/F0 and different Cu^2+^ concentrations (CDs, 0.05 mg/mL, excitation wavelength at 360 nm, emission wavelength at 430 nm). The inset was the linear plot of the ratiometric fluorescence intensity to the Cu^2+^ concentration in the range of 0–1.72 μM; (**c**) Fluorescence responses of the CDs at 430 nm (CDs, 0.05 mg/mL, excitation wavelength at 360 nm, emission wavelength at 430 nm) with different ions (860 μM) and (**d**) schematic preparation of N, S-CDs and its detection for Cu^2+^ and GSH Adapted with the permission of ref. [103].

In addition to mercury, cellulose derived carbon dots were also used for the detection of other heavy metals such as Iron (III) (Fe^3+^). The group of Fan developed a nitrogen-doped carbon dots (N-CDs) prepared by a hydrothermal method using microcrystalline cellulose as the carbon source and polyethylenimine as the nitrogen source. The ratio between microcrystalline cellulose and polyethylenimine added was studied showing that it exhibits a great influence on the fluorescence behavior and the highest performance was attributed to the 1:1 ratio. The fluorescence intensity of the proposed material showed a good linear relationship with Fe^3+^ concentrations from 0 to 14 ppm achieving a limit of detection of 0.21 ppm. Interestingly, the proposed sensor was successfully used in practical application using tap and pond water showing a great recovery ranging from 98.25 to 102.75% with a low standard deviation [101]. Another method to obtain CDs is through the valorisation of agriculture waste. For this, Gao et al. transformed coconut petiole residues, naturally rich in cellulose, into CDs to develop a sensor for Fe^3+^ detection in water. These CDs emit strong blue fluorescence under ultraviolet radiation. Upon Fe^3+^ binding, the material undergoes a synergetic effect of static and dynamic quenching of its fluorescence. It also exhibits sensitivity within a linear range from 0.005 to 0.2 mM and a detection limit of 2.3 µM. The proposed sensor was also tested using tap and lake water as a real sample and showed good recovery demonstrating its feasibility to detect Fe^3+^ in real environmental water [102]. Cellulose derived carbon dots were used to detect copper ions (Cu^2+^) as reported by Wang et al. Using an ionothermal approach in a deep eutectic solvent, which act as a solvent and a dopant, nitrogen- and sulfur-co-doped carbon dots were successfully synthesized. Nitrogen and sulfur co-doping enhances fluorescence quantum yield and improve interaction with target analyte. Once exposed to copper, the fluorescence of CDs is selectively quenched by Cu^2+^ ions through complexation mechanism. As evidenced in Figure 8, the fluorescent probe can selectively detect Cu^2+^ with a high sensitivity of 23.4 nM [103]. Also, the group of Issa et al. successfully detect Cu^2+^ ions using nitrogen-doped carbon dots (N–CDs) from lignocellulosic waste [104]. The highly fluorescent N–CDs was obtained from the carboxymethylcellulose of oil palm empty fruit bunches and linear-structured polyethyleneimines. The fluorescent probe was tested within the range 1–30 µM, with a detection limit of 0.93 µM. The fluorescent probe was successfully applied for the detection of Cu^2+^ in real water.

### 6.2. Detection of Pesticides

**Figure 9:**
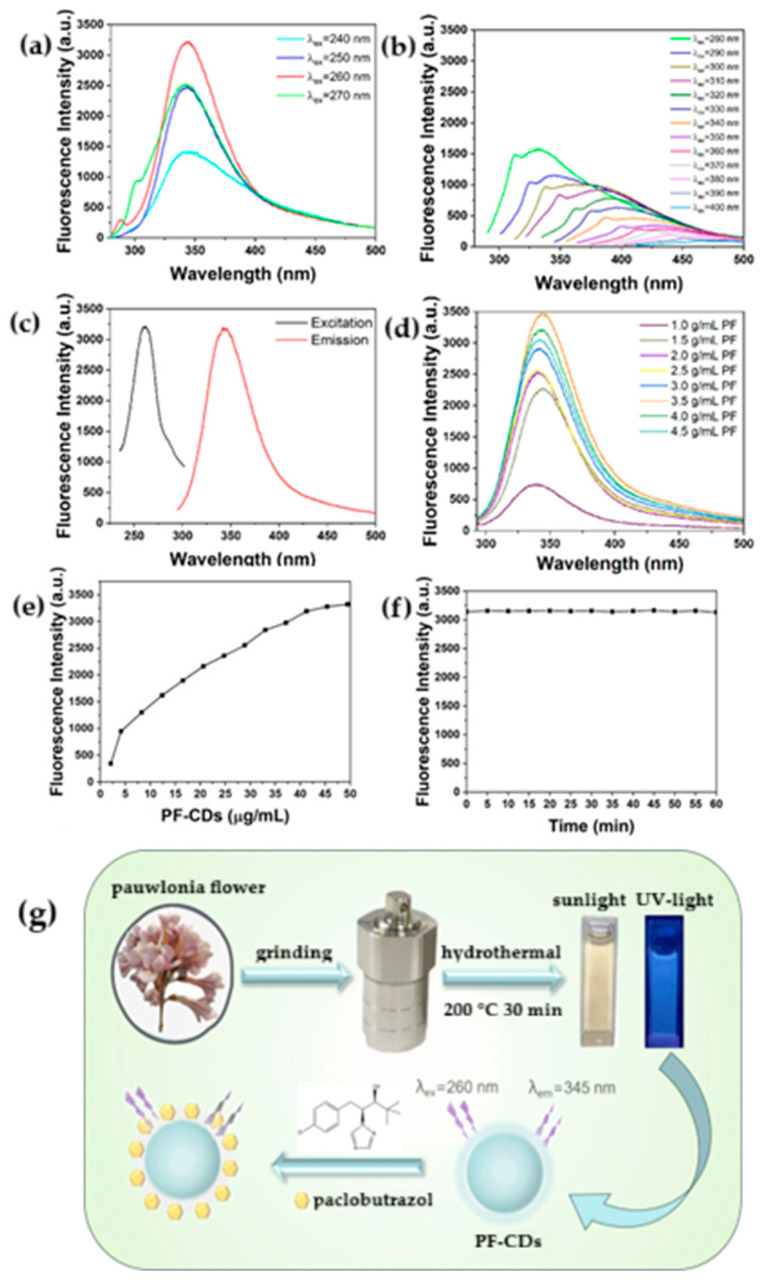
(**a**,**b**) Fluorescence spectra of PF-CDs under excitation wavelengths at 240–400 nm, (**c**) Excitation and emission spectra of PF-CDs, (**d**) Fluorescence spectra of PF-CDs with different precursor concentrations, (**e**) Fluorescence intensity of PF-CDs with different PF-CDs concentrations, (**f**) Fluorescence intensity of PF-CDs under irradiating for 2 h, (**g**) The preparation process and detection principle of PF-CDs. (Adapted with the permission from ref. [105]).

Carbon dots derived from cellulose were also used for pesticides detection. The group of Zhang synthesized CDs via a simple, eco-friendly hydrothermal method using paulownia flower, naturally rich on cellulose, as the carbon source [105]. As evidenced in Figure 9, the CDs exhibit strong blue fluorescence behaviour. The fluorescence intensity of the paulownia flower-derived CDs is quenched efficiently by paclobutrazol in the range from 0.78 to 18.75 μg/L, enabling sensitive detection. The LOD was around 0.005 μg/L. The quenching mechanism is mainly attributed to complex formation between paclobutrazol molecules and the surface groups on the CDs. To test the reliability and applicability of their fluorescent probe for detecting paclobutrazol pesticides in real samples, they used the standard addition method in apple juice samples. The recovery rate from 94.06–108.56% indicate the high accuracy of the CDs-based sensor in complex matrices. The study found that both EAS2B (human normal lung epithelial) and A549 (human lung carcinoma epithelial) cells exhibit over 90% viability when incubated with PF-CDs at concentrations up to 0.1 mg/mL, indicating minimal toxicity.

This confirms the potential for safe use of PF-CDs in biological systems. PF-CDs were, also, able to effectively penetrate the cell membrane of BMDM (mouse bone marrow-derived macrophage) cells during incubation, as evidenced by the detection of blue fluorescence under a fluorescence microscope. This highlights the feasibility of using PF-CDs as fluorescent probes for biological imaging applications. Indeed, PF-CDs exhibited stable, photostable, and water-soluble fluorescence properties, the remaining fluorescence intensity of PF-CDs significantly unchanged even after 2 h of continuous irradiation.

### 6.3. Detection of Other Pollutants

Sugarcane bagasse is one of the most well-known solid contaminants from agricultural waste, causing serious problems worldwide. Simple techniques could transform this agricultural waste into valuable materials. Alfi et al. first isolated cellulose from this waste, obtaining fibrous cellulose in the form of a white powder. This latter product then underwent further processing leading to cellulose diacetate, followed by a hydrothermal method and ammonium hydroxide treatment to produce nitrogen-doped carbon particles (NCD). NCD were used as a fluorescent probe for tetracycline detection. The sensor works based on fluorescence quenching. In the presence of tetracycline, the fluorescence intensity of the carbon dots changes linearly in the range from 0–110 μM, allowing for easy quantitative detection. The detection limit was set at 0.01 μΜ [106]. Cellulose-derived CDs were also used by the group of Meng for the detection of silver ions (Ag^+^). The CDs were produced from carboxymethyl cellulose (CMC). They exhibited bright yellow fluorescence and showed high selectivity toward Ag^+^. In order to test the sensing performance of these CDs several Ag+ concentrations were added in the range from 0 to 200 μΜ. The detection limit (LOD) was calculated to be 0.01 μM. Selectivity of this sensor was evaluated by adding 15 metal ions, 9 anions and 14 organic small molecules at the same concentrations to the CDs solutions. Only the addition of Ag+ induced a significant fluorescence quenching, while other ions and molecules had minimal impact on the fluorescence intensity proving that this sensor is valid for practical use. Tap water, Yitong river water, and south lake sample water were used as real samples. The recoveries were obtained ranging from 96.97% to 104.75%, demonstrating good accuracy and reliability of the proposed method [107].

Taken together, these studies demonstrate the remarkable versatility and effectiveness of cellulose-derived carbon dots in detecting a wide spectrum of environmental contaminants. However, despite the impressive laboratory performance, several challenges still hinder their translation into real-world sensing systems, as discussed in the following section.

## 7. Challenges, Limitations and Future Perspectives

Although cellulose-derived CDs have demonstrated remarkable potential in environmental sensing, several critical challenges still hinder their widespread adoption. One of the most persistent issues is the batch-to-batch reproducibility of synthetic procedures. Even minor variations in precursor source, cellulose pretreatment, reaction conditions, or post-synthetic purification can lead to substantial differences in particle size distribution, surface functionalization, and optical properties, ultimately affecting sensing performance [57,108]. The absence of standardized protocols for synthesis and characterization exacerbates this problem: currently, different laboratories employ diverse synthetic routes, detection methods, and analytical criteria, making it difficult to directly compare results and establish universal benchmarks [54]. Future efforts should therefore focus on establishing standardized synthesis and characterization protocols, including reference materials, calibration methods, and agreed analytical metrics, to ensure data reproducibility and comparability across laboratories. Another significant concern is the stability and durability of CD-based sensing systems under real environmental conditions. Laboratory studies typically report excellent sensitivity and selectivity, but field deployment often introduces variables such as fluctuating ionic strength, the presence of natural organic matter, photobleaching under UV exposure, and long-term storage issues, which can compromise sensor reliability [54]. Scalability also represents a major bottleneck. While cellulose is abundant, inexpensive, and renewable, the conversion of biomass into reproducible high-quality CDs requires carefully controlled processes that are not yet economically viable at industrial scale [57]. The additional steps required to immobilize CDs onto substrates or incorporate them into composite matrices for device fabrication further raise production costs. Thus, moving from proof-of-concept demonstrations to commercially competitive platforms remain challenging. To address these challenges, future research should focus on developing advanced sustainable synthetic strategies aligned with circular economy and zero-waste principles. This includes valorising agricultural residues, recycling solvents, and coupling CD production with biorefinery processes [53]. Such approaches not only minimize environmental impact but also enhance the economic feasibility of large-scale CD production. In parallel, the integration of cellulose-derived CDs into biodegradable polymer matrices such as cellulose acetate, chitosan, or polylactic acid represents a feasible route toward flexible and eco-friendly sensing devices. These composites combine the optical activity of CDs with the mechanical stability and recyclability of biopolymers, enabling scalable fabrication of sustainable membranes and films for environmental monitoring and wearable applications. Another promising avenue is the engineering of hybrid materials and nanocomposites, where cellulose-derived CDs are combined with polymers, metal nanoparticles, or 2D nanostructures. These hybrid systems can synergistically enhance selectivity, sensitivity, and multifunctionality, making them suitable for advanced sensing platforms [56]. CD–cellulose composites hold strong promise for flexible membranes and films that merge sustainability with functionality. Moreover, the development of multimodal sensing systems, such as photoelectric or ratiometric dual-response platforms, offers an innovative strategy to improve analytical accuracy and robustness. By integrating fluorescence and electrochemical transduction within a single device, these systems can provide self-calibration, reduce false positives, and enable simultaneous detection of multiple contaminants. The integration of CDs into flexible electronics and Internet of Things (IoT)-based sensing devices is another frontier with high potential. Wearable or portable CD-based sensors could enable real-time monitoring of contaminants in water, air, or soil, with wireless data transmission and on-site decision-making [10]. Achieving this will require innovations not only in optical sensing but also in electrochemical and photoelectrochemical transduction. Additionally, future studies should incorporate quantitative life-cycle and energy-efficiency analyses to evaluate the true sustainability of CD synthesis. Defining measurable indicators, such as energy demand, carbon footprint, and solvent recyclability, will be essential to guide greener industrial-scale processes. Finally, as the field matures, attention must be given to regulatory frameworks, safety evaluation, and commercialization pathways. Comprehensive toxicological studies, standardization of testing protocols, and life-cycle assessments will be essential to facilitate regulatory approval and industrial uptake [7]. The definition of clear guidelines for environmental and human safety will determine the pace of translation from laboratory research to real-world applications.

In conclusion, cellulose-derived carbon dots represent a versatile and sustainable nanomaterial platform that has already demonstrated great potential in environmental sensing. Advances in green synthetic strategies have enabled the valorization of cellulose as a renewable precursor, yielding CDs with tunable optical and electronic properties and excellent compatibility with aqueous systems [55,109]. Significant progress has been made in detecting heavy metals, antibiotics, pesticides, and organic pollutants, highlighting the versatility and applicability of these materials in addressing urgent environmental challenges [11]. Nevertheless, the field still faces non-trivial limitations related to reproducibility, stability, scalability, and device integration. Overcoming these barriers will require interdisciplinary collaboration, bringing together expertise from materials science, chemistry, engineering, and regulatory sciences. Standardized protocols, multimodal sensor architectures, and sustainable composite designs will be crucial steps toward the next generation of cellulose-derived CD technologies. Looking ahead, the combination of hybrid material engineering, flexible device integration, and circular economy-driven strategies promises to transform cellulose-derived CDs from laboratory curiosities into next-generation sustainable sensor technologies. Their ability to merge green chemistry principles with high-performance sensing makes them strong candidates for real-world deployment in environmental monitoring systems of the near future.

## Figures and Tables

**Table 1:** Summary of Advantages and Limitations of Cellulose as a Renewable Precursor for CDs Synthesis.

Features	Advantages	Limitations	Ref.
Abundance & Renewability	- Most abundant organic polymer worldwide. - Derived from plants, bacteria, algae, and waste biomass. - Renewable, low-cost, and globally available feedstock.	- Variability in composition depending on origin (wood, agricultural residues, bacterial cellulose). - Heterogeneity in crystallinity and polymerization degree.	[26,42]
Eco-Friendliness	- Valorization of waste biomass supports circular economy. - Reduces environmental impact and waste disposal problems.	- Biomass pretreatment may require harsh chemicals (acids, bases, ionic liquids). - Energy-intensive processes increase costs and may offset sustainability.	[42,53]
Chemical Functional Groups	- Rich in hydroxyl (–OH), carboxyl (–COOH), aldehyde groups. - Facilitates doping, surface passivation, and functionalization.	- Retention of functional groups depends on synthesis control. - Harsh carbonization may reduce surface functionality, lowering PL yield.	[26,54]
Biocompatibility	- CDs from cellulose usually show low cytotoxicity. - Excellent water dispersibility and biocompatibility for bio- and eco-sensing.	- Properties vary with precursor purity and synthesis route. - Potential cytotoxic effects if residual impurities remain.	[11,52]
Versatility	- Cellulose can be processed into nanocellulose, fibers, films, or hydrogels before CD synthesis. - Enables control over morphology and optical properties.	- Waste-derived cellulose requires complex pretreatment and purification. - Extraction routes can reduce scalability and reproducibility.	[55,56]
Carbon Yield	- Abundant precursor guarantees low raw material cost.	- Low carbon content (~44 wt% in pure cellulose). - Lower CD yields compared to other biomass or fossil-based precursors.	[57,58]
Reproducibility	- —	- Heterogeneity due to presence of lignin/hemicellulose and impurities. - Leads to variability in PL properties, size distribution, and sensing performance.	[7,54]

**Table 2:** Comparative Analysis of Cellulose-Derived CD Synthesis Methods.

Method	Description	Advantages	Critical Issues	Ref.
**Hydrothermal Solvothermal**	Heating cellulose suspensions (150–300 °C) in sealed autoclave; solvothermal involves aqueous–organic solvents	✓ Widely used and well established ✓ One-pot process (hydrolysis, carbonization, passivation) ✓ Doping possible by precursor choice ✓ Versatile surface functionalities ✓ Suitable for photocatalysis and sensing	✓ Requires high temperature and pressure ✓ Relatively long reaction times ✓ Energy-intensive ✓ Risk of heterogeneous particle size if poorly controlled	[75,76]
**Microwave-Assisted**	Rapid heating of cellulose or derivatives under microwave irradiation (300–900 W, 5–20 min)	✓ Very fast synthesis ✓ High energy efficiency ✓ Narrow size distribution ✓ High QY (~30%) achievable ✓ Tunable surface chemistry ✓ Good antimicrobial/antioxidant properties	✓ Limited scalability (lab-scale equipment) ✓ Risk of non-uniform heating in larger batches ✓ Requires pre-treatment of cellulose	[65,69]
**Pyrolysis/Combustion**	Thermal decomposition (300–600 °C) in inert atmosphere (pyrolysis) or open-air (combustion)	✓ Simple, solvent-free ✓ Scalable and cost-effective ✓ High carbonization efficiency ✓ Direct heteroatom doping possible	✓ Requires high operational T (≥ 300 °C) ✓ Often low or variable QY (11–29%) ✓ Risk of graphitic impurities ✓ Requires post-purification	[70,71]
**Ultrasound-Assisted**	Cavitation bubbles create local high T/P, degrading cellulose	✓ Mild, green process ✓ Low T operation, short reaction times ✓ Abundant surface functionalities ✓ Good photostability ✓ Lower energy consumption	✓ CDs may show lower QY than pyrolysis-derived ✓ Requires optimization of sonication conditions ✓ Often co-solvents needed	[72,74]
**Other green methods (e.g., DES, enzymatic hydrolysis)**	Deep eutectic solvents (DES) or enzymes under mild T (~110 °C)	✓ Very mild, environmentally friendly ✓ DES recyclable ✓ Strong fluorescence (broad emission) ✓ Potential for sensor inks and flexible films	✓ Still emerging, less standardized ✓ Scalability yet to be proven	[73]

**Table 3:** Functionalization Impact on Electrochemical Properties of CDs.

Functionalization Strategy	Effect on Properties	Applications in Electrochemical/Sensing	(Ref.)
Surface Passivation (PEG, EDA, Citric Acid)	Reduces surface traps, increases fluorescence, improves stability and dispersibility.	Enhanced electrochemical stability in aqueous media; better signal reproducibility in biosensing platforms.	[88,89,90]
N-Doping	Introduces electron-rich sites, increases QY, improves electron transfer.	Sensitive detection of electron-deficient analytes; enhanced electrochemical current response.	[91]
P-Doping	Increases binding affinity to certain metal ions; stabilizes charge transfer.	Selective sensing of metal ions; improved redox activity.	[92]
S-Doping and N, S Co-Doping	Introduces mid-gap states; red-shifted emission; generates redox-active sites.	Enhanced antioxidant and electrochemical sensing; tunable emission for multiplexed detection.	[93]
Fe-Doping	Creates additional catalytic active sites; improves charge transfer kinetics.	Electrochemical detection of Cu^2+^; catalytic degradation of urea.	[94]
Cu-Doping	Enhances dual-mode sensing via FRET and inner filter effect; improves selectivity.	Dual-mode fluorescence + electrochemical sensing of Cu^2+^.	[95]
Surface Functionalization with Amine Groups (-NH_2_)	Facilitates electron transfer via hydrogen bonding and chelation with nitroaromatics.	Electrochemical/fluorescence detection of nitroaromatic explosives.	[96]
Surface Functionalization with Thiol Groups (-SH)	Strong chelation with Hg^2+^; improves quenching efficiency.	Ultrasensitive detection of Hg^2+^ via fluorescence and electrochemical methods.	[97]
Surface Carboxyl Groups (-COOH)	Improves dispersibility and electron transfer via hydrogen bonding.	Electrochemical sensing of amines and nitro compounds; improved biocompatibility.	[99,100]

**Table 4:** Electrochemical performance of biomass and green processes CDs based sensors for the detection of several analytes.

Field	Analyte	Source	Doping	Detection Range (µM)	Detection Limit (µM)	Real Sample	Ref.
**Heavy metals**	Hg^2+^	Cellulose Hydrogel	Oxygen, Nitrogen, Sulfur Doping	0.2–100	~0.2	-	[17]
	Hg^2+^	Carboxymethyl Nanocellulose	Amine, Nitrogen Doping	0–100	8.29	-	[100]
	Hg^2+^	Bamboo Cellulose	None	5 × 10^−4^–1 × 10^−3^	5.16 × 10^−3^	Tap water Industry sample	[10]
	Fe^3+^	Microcrystalline Cellulose	Nitrogen Doping (Polyethylenimine)	0–250.72	3.76	Tap and pond water	[101]
	Fe^3+^	Coconut Petiole Residues	None	5–200	2.3	Tap and lake water	[102]
**Heavy metals**	Cu^2+^	Cellulose (via Ionothermal Approach)	Nitrogen & Sulfur Doping	0–1.7	0.0234	-	[103]
	Cu^2+^	Lignocellulosic Waste (Oil Palm)	Nitrogen Doping (Carboxymethylcellulose & Polyethylenimine)	1–30	0.93	Real water samples	[104]
**Pesticide**	Paclobutrazol	Paulownia Flower (Rich in Cellulose)	None	2.65–63.83	0.017	Apple juice samples	[105]
**Other pollutants**	Tetracycline (Antibiotic)	Sugarcane Bagasse (Rich in Cellulose)	Nitrogen Doping	0–110	0.01	-	[106]
	Silver Ions (Ag^+^)	Cellulose (carboxymethyl cellulose)	None	0–200	0.01	Tap water, Yitong river water, South lake sample water	[107]

## Data Availability

Not applicable.
